# A bibliometric analysis of Prader-Willi syndrome from 2002 to 2022

**DOI:** 10.1515/med-2024-1058

**Published:** 2024-11-28

**Authors:** Cai-Xia Yang, Xiu-Yun Jiang, Xiao-Hong Li

**Affiliations:** Department of Statistics and Medical Record Management, Shandong Provincial Hospital Affiliated to Shandong First Medical University, Jinan, Shandong, 250021, China; Department of Endocrinology, Shandong Provincial Hospital Affiliated to Shandong First Medical University, Jinan, Shandong, 250021, China; International Medical Service Department, Shandong Provincial Hospital Affiliated to Shandong First Medical University, Jinan, Shandong, 250021, China

**Keywords:** Prader-Willi syndrome, ghrelin, bibliometric analysis, CiteSpace, VOSviewer

## Abstract

**Background:**

Prader-Willi Syndrome (PWS) is a rare disorder that was initially documented by Prader and Willi in 1956. Despite significant advancements in the understanding of PWS over recent decades, no bibliometric studies have been reported on this field. We aimed to analyze and explore the research trends and hotspots of PWS using a bibliometric analysis to understand the future development of basic and clinical research.

**Methods:**

The literature regarding PWS was retrieved from the Web of Science Core Collection Science Citation Index Expanded (SCI-Expanded) database. Data were extracted from the articles or review articles, and analyzed using CiteSpace and VOSviewer software.

**Results:**

A total of 1,895 related studies have been published in 64 countries or regions. The United States has published the most articles, followed by the United Kingdom, Italy, Netherlands, and France. University of Florida (The United States), University of Kansas (The United States), University of Alberta (Canada), University of Cambridge (the United Kingdom), and Dutch Growth Research Foundation (Netherlands) were the top five most productive institutions. Butler, Merlin G. and his colleagues have made the most outstanding contributions in the field of PWS research. Keyword co-occurrence analysis showed that genomic imprinting, uniparental disomy, obesity, hyperphagia, hypothalamus, growth hormone treatment, and ghrelin appeared with the higher frequency. Furthermore, oxytocin, magel2, and management were the latest bursts keywords.

**Conclusion:**

Our findings indicated that genetic mechanism, diagnose, and emerging therapies will be the hotspots and frontiers in PWS research.

## 1 Introduction

Prader-Willi syndrome (PWS) is a rare disorder due to the absence of paternal allele expression in the 15q11-13 region [1]. PWS affects approximately one out of every 15,000–20,000 people, with a global estimated 400,000 cases. Multiple genetic mechanisms have been identified as underlying causes: the deletion of 15q11-q13 occurs in 60%, maternal uniparental disomy 15 (UPD15) in 36%, and imprinting center defects in 4% [2]. The hallmark clinical manifestations of PWS encompass severe neonatal hypotonia, early hyperphagia leading to morbid obesity, growth retardation, hypogonadism, learning disabilities, and behavioral challenges [1,3]. Clinical diagnosis primarily relies on these symptoms and is subsequently confirmed through genetic testing [4].

PWS was first described by two Swiss pediatric endocrinologists Andrea Prader and Heinrich Willi, as well as internist Alexis Labhart in 1956. This collaboration published the first article on this syndrome and helped to understand the causes of death in PWS patients, as well as proposing therapies to improve overall quality of life. Currently, there is still no cure for PWS, and a multidisciplinary approach throughout the lifespan is necessary, consisting of early diagnosis, collaboration with multidisciplinary teams, growth hormone (GH) therapy, dietary control, and enhanced understanding of behavioral and psychiatric aspects [5]. Despite significant advancements in PWS over recent decades, comprehensive reports that offer researchers an intuitive overview and insights into research trends and hotspots are still absent.

Bibliometrics is a quantitative analysis approach in knowledge fields [6]. It can intuitively visualize the knowledge structure and delve into research trends, hotspots, and frontiers [6,7]. Citespace software [8] and VOSviewer software [9] are useful bibliometric analysis tools. This study aimed to analyze research hotspots and frontiers from 2002 to 2022 in the field of PWS to offer an intuitive visualization and comprehensive understandings for guiding future research.

## 2 Methods

### 2.1 Search strategy and data collection

The Web of Science Core Collection (WoSCC) SCI-Expanded database was used to comprehensively search publications. The Medical Subject Headings (Mesh) of “PWS” was retrieved. Our search strategy includes the following entry terms: Prader Willi Syndrome, Syndrome and Prader-Willi, Labhart-Willi Syndrome, Labhart Willi Syndrome, Syndrome and Labhart-Willi, Labhart-Willi-Prader-Fanconi Syndrome, Labhart Willi Prader Fanconi Syndrome, Syndrome and Labhart-Willi-Prader-Fanconi, Willi-Prader Syndrome, Syndrome and Willi-Prader, Willi Prader Syndrome, Prader Labhart Willi Syndrome, Prader-Labhart-Willi Syndrome, Syndrome and Prader-Labhart-Willi, Royer Syndrome, Syndrome and Royer, Royer’s Syndrome, Royers Syndrome, and Syndrome and Royer’s. The advanced search mode with title (TI), abstract (AB), and author keywords (DE) [10,11] were used. Only English articles and review articles published between January 1, 2002 and December 31, 2022 were included in this analysis. The meeting abstracts, editorial materials, corrections, letters, retractions, and proceedings papers were excluded. The full records along with their cited references were extracted on July 17, 2023, and saved as plain text files using Clarivate Analytics.

### 2.2 Data analysis

Records retrieved from WoSCC SCI-Expanded were analyzed by Microsoft Excel 2010, VOSviewer version 1.6.19 (Leiden University, Leiden, Netherlands), and CiteSpace version 6.2.R4 (Drexel University, Philadelphia, PA, USA). A polynomial regression model was fitted using Microsoft Excel 2010 to predict the growth trend of the papers.

VOSviewer was used to analyze the institutions, authors/co-cited authors, journals/co-cited journals, author keywords, all keywords, keywords plus with minimum number of documents of analyses object being 10, 10/100, 5/100, 5, 15, 15, respectively. CiteSpace was used to analyze countries/regions network and references visualizations including co-citation, clustering, timeline, keyword burst detection. Duplicates were conducted with CiteSpace. The settings of all analysis were followed: time slicing from January 2002 to December 2022, years per slice 1, *k* = 25, pruning = none, and cluster labels generated from the keywords of citing articles.

## 3 Results

### 3.1 Research trends in PWS

**Figure 1:**
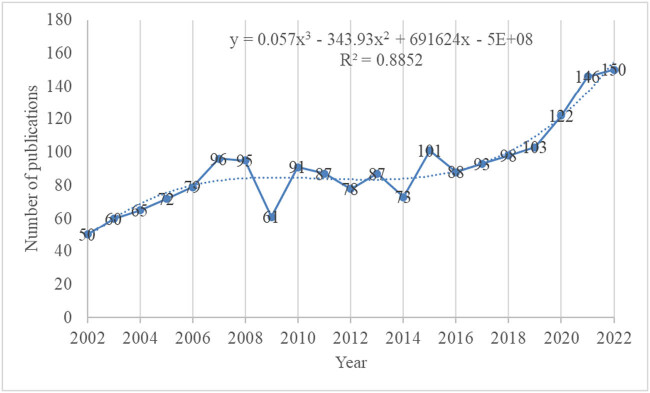
Growth trends of publications on Prader-Willi syndrome from 2002 to 2022.

Between 2002 and 2022, a total of 1,895 papers related to PWS were published and indexed in the WoSCC SCI-Expanded. As shown in Figure S1, among these publications, original articles account for 86% and comments account for 14%. The number of published papers per year and a fitted polynomial regression model are presented in Figure 1. This analysis revealed a consistent upward trend in the number of publications, increasing from 50 in 2002 to 150 in 2022. This corresponded to an annual growth rate of 5.7%. Notably, the period from 2016 to 2022 exhibited a particularly rapid increase, with an annual growth rate of 9.3%.

### 3.2 Countries/regions and institutions

**Figure 2:**
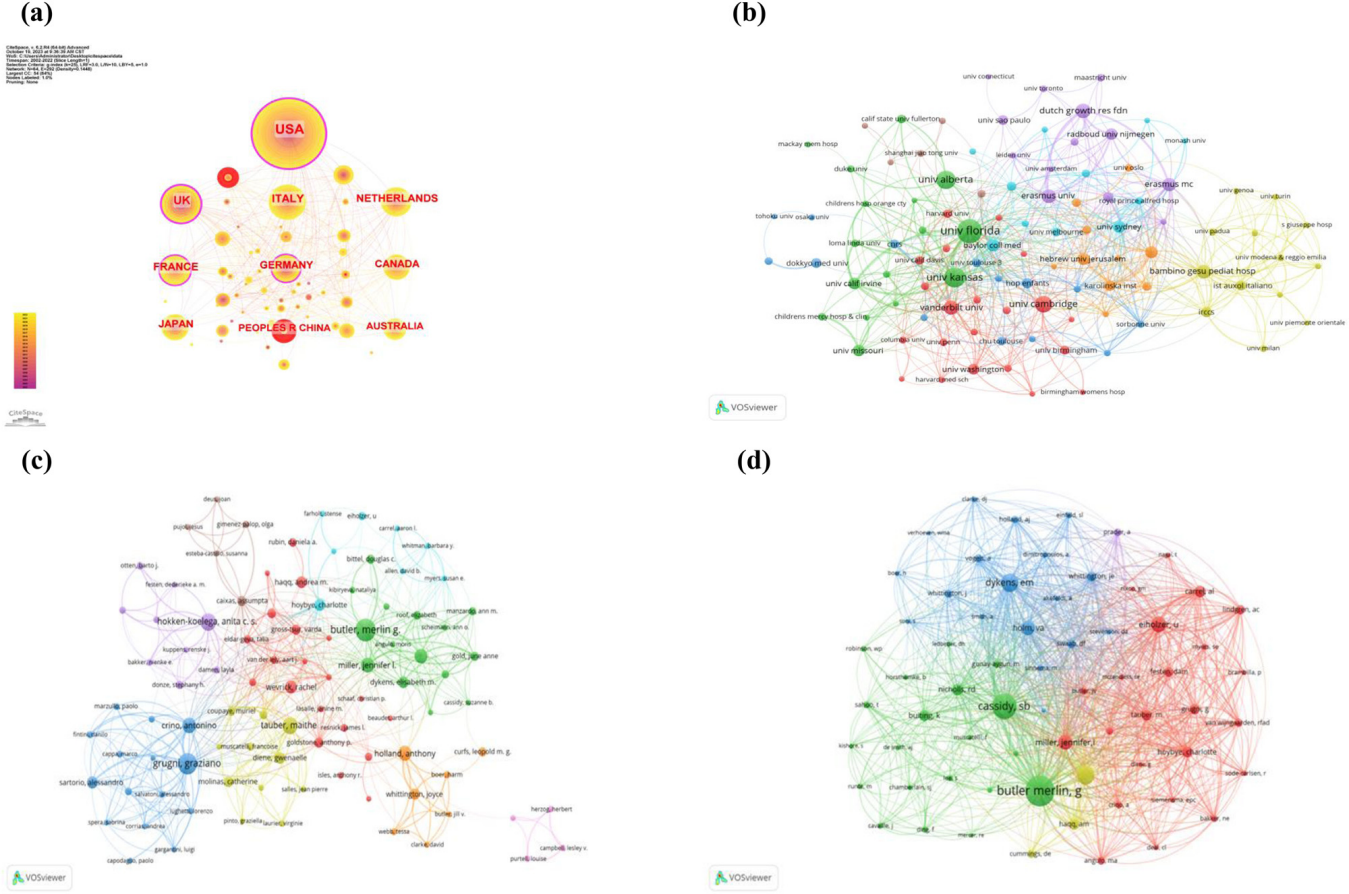
(a) Collaboration networks between countries/regions, the thickness of a purple trim represented high betweenness centrality, the presence of red tree ring revealed burstness of count. (b) Institutions collaboration networks. (c) Authors networks. (d) Co-cited authors’ networks.

Countries/regions collaboration networks are shown in Figures 2a and S2a. There were 64 countries/regions published papers in the field of PWS research. The largest contributor was USA (622), followed by UK (204), Italy (185), Netherlands (158), and France (150). The betweenness centrality of USA was 0.37, followed by UK (0.33), France (0.14), and Germany (0.11) (Table S1). Nodes with high betweenness centrality (>0.1) in the networks are deemed as key hubs.

Institutions collaboration networks are shown in Figures 2b and S2b. University of Florida in USA, University of Kansas in USA, University of Alberta in Canada, University of Cambridge in UK, Dutch Growth Research Foundation in Netherlands were the top five most productive institutions (Table S1). University of Florida in USA had a greater influence than other institutions in the field of PWS research.

### 3.3 Authors and co-cited authors

Co-authorship networks are presented in Figure 2c. Out of 7,632 authors 104 published at least ten papers of PWS; 94 items and 2,619 total link strength were contained in this network. Co-cited authors’ networks are presented in Figure 2d. Out of 30,249 authors, 65 have been cited at least 100 times. The top ten most productive authors and co-cited authors are shown in Table S2. Butler, Merlin G. and his colleagues have published 99 papers with 1,487 citations, further highlighting their enormous influence in this field.

### 3.4 Journals and co-cited journals

There were 605 journals and 6,398 co-cited journals related to PWS. The visualization maps of journals and co-cited journals are presented in Figure S3. The most productive and influential journals are shown in Table 1. The American Journal of Medical Genetics Part A exhibited the most prolific outputs (144, 7.6%). The Journal of Clinical Endocrinology and Metabolism ranked second (87, 4.6%) with the highest impact factor (IF = 5.8) within the top ten journals. In the light of the Journal Citation Reports in 2022, a significant majority of the journals were classified into the prestigious Q1 and Q2 categories, thereby further emphasizing their high-quality content and substantial impact on the field. As illustrated in Table 1, all ten co-cited journals have garnered over 1,000 citations each. The Journal of Clinical Endocrinology and Metabolism stood out with an impressive number of citations (4,820), followed by American Journal of Medical Genetics Part A (2,514), Human Molecular Genetics (2,083), Nature Genetics (1,851), and American Journal of Medical Genetics (1,803). Notably, 90% of these co-cited journals are categorized within Q1 and Q2 regions. Nature holds the highest impact factor (IF = 64.8).

**Table 1:** Top ten productive journals and co-cited journals

Rank	Journals	Count (%)	IF (2022)	JCR	Co-cited journals	Citations	IF (2022)	JCR
1	Am J Med Genet A	144 (7.6)	2.0	Q3	J Clin Endocrinol Metab	4,820	5.8	Q1
2	J Clin Endocrinol Metab	87 (4.6)	5.8	Q1	Am J Med Genet A	2,514	2.0	Q3
3	J Pediatr Endocrinol Metab	44 (2.3)	1.4	Q4	Hum Mol Genet	2,083	3.5	Q2
4	Clin Endocrinol (Oxf)	39 (2.1)	3.2	Q3	Nat Genet	1,851	30.8	Q1
5	J Clin Med	39 (2.1)	3.9	Q2	Am J Med Genet	1,803	3.7	Q2
6	Hum Mol Genet	34 (1.8)	3.5	Q2	Am J Hum Genet	1,496	9.8	Q1
7	Orphanet J Rare Dis	34 (1.8)	3.7	Q2	Pediatrics	1,489	8.0	Q2
8	Plos One	26 (1.4)	3.7	Q3	J Med Genet	1,427	4.0	Q1
9	J Pediatr	22 (1.2)	5.1	Q1	J Intellect Disabil Res	1,276	3.6	Q1
10	Eur J Hum Genet	21 (1.1)	5.2	Q2	Nature	1,222	64.8	Q1

Abbreviations: IF, impact factor; JCR, Journal Citation Reports.

### 3.5 References co-citation analysis

#### 3.5.1 Most cited papers

**Figure 3:**
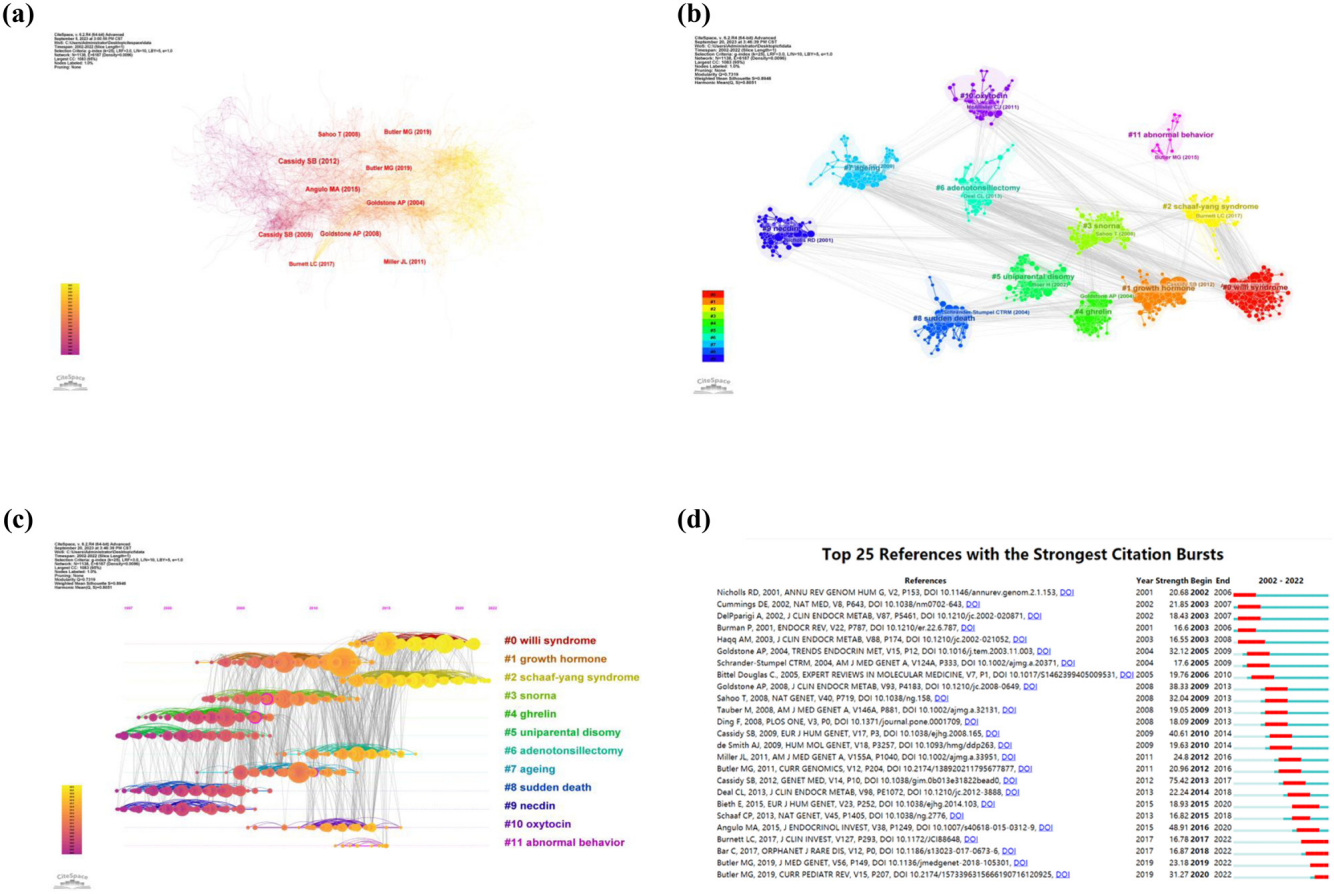
(a) References co-citation map, with the ten most cited references marked. (b) Cluster networks. (c) Timeline view. (d) Top 25 references with the strongest citation bursts.

A total of 42,024 valid references were analyzed utilizing the Citespace software, and merged network included 1,138 nodes and 6,187 links. References co-citation networks are shown in Figure 3a. The top ten most frequently co-cited references are presented in Table S3. The most cited paper, “PWS,” is frequently cited and holds significant popularity and influence within its research field [12]. “PWS” published by Cassidy et al. in the Genetics in Medicine [1] emerged as the most co-cited paper with 159 co-cited counts. The second most cited paper was by Angulo et al., in the Journal of Endocrinological Investigation [13] with 105 co-cited counts. The third most cited paper is “PWS” published by Cassidy and Driscoll in the European Journal of Human Genetics [14] with 90 co-cited counts.

#### 3.5.2 Clusters of research

The 12 major clusters and their most representative reference are shown in Figure 3b. The 12 clusters were marked as “ #0 Willi syndrome,” “ #1 growth hormone,” “#2 Schaaf-Yang syndrome,” “#3 snorna,” “#4 ghrelin,” “#5 uniparental disomy,” “#6 adenotonsillectomy,” “#7 ageing,” “#8 sudden death,” “#9 necdin,” “#10 oxytocin,” and “#11 abnormal behavior.”

Timeline view of references is shown in Figure 3c, indicating the evolution of research topic trends regarding PWS. Research trends can be broadly divided into three major phases. The first phase approximately came from 1997 to 2008, and themes mainly consisted of ghrelin and UPD. The second phase approximately started from 2003 to 2015, and themes included GH and oxytocin. The third phase approximately began from 2012 to 2022, and themes were Willi syndrome and Schaaf-Yang syndrome. The top 25 references with the strongest bursts are presented in Figure 3d, demonstrating their importance in the field of PWS.

### 3.6 Keywords co-occurrence analysis

**Figure 4:**
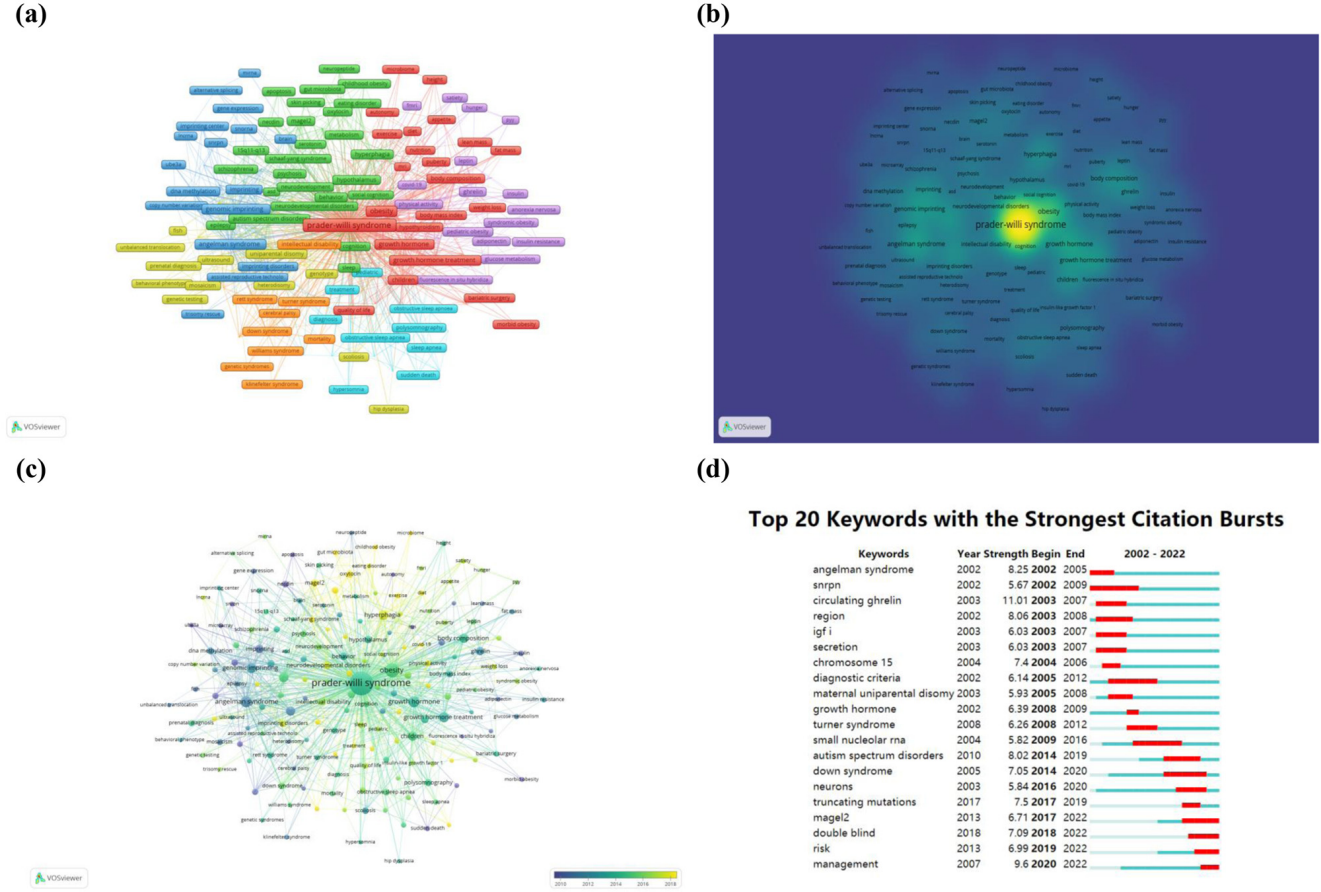
(a) Author keywords co-occurrence network visualization, different colors represent different clusters. (b) Author keywords density visualization. (c) Author keywords overlay visualization and different colors represent different years. (d) Top 20 keywords with the strongest citation bursts.

The top five keywords with the most occurrences were PWS (1,040 occurrences), obesity (188 occurrences), growth hormone (94 occurrences), Angelman syndrome (86 occurrences), growth hormone treatment (69 occurrences) (Table 2). The results of keywords co-occurrence networks are shown in Figure 4. Three main research clusters of PWS could be summarized as (1) genetic mechanism: genomic imprinting, imprinting, UPD; (2) clinical aspects: obesity, hyperphagia, ghrelin, oxytocin, autism spectrum disorders, intellectual disability, hypothalamus; and (3) treatment options: GH deficiency, GH treatment (Figure 4a and b). The keywords such as magel2, oxytocin, and rare diseases appeared recently, showing that these themes may be the research frontiers (Figure 4c). The results of keywords burst detection showed that magel2 (2017–2022), double blind (2018–2022), risk (2019–2022), and management (2020–2022) were the latest burst keywords (Figure 4d). Similar results were obtained from all keywords (Figure S4) and keywords plus (Figure S5) analysis.

**Table 2:** Top 20 most author keywords

Rank	Author keywords	Counts	Total link strength	Rank	Author keywords	Counts	Total link strength
1	Prader-Willi syndrome	1,040	1,828	11	Body composition	48	118
2	Obesity	188	454	12	Imprinting	38	91
3	Growth hormone	94	236	13	Ghrelin	37	93
4	Angelman syndrome	86	240	14	Growth hormone deficiency	30	76
5	Growth hormone treatment	69	154	15	Behavior	28	80
6	Genomic imprinting	61	148	16	Intellectual disability	27	63
7	Autism spectrum disorders	59	150	17	Genetics	26	70
8	Hyperphagia	57	137	18	Hypothalamus	25	69
9	Children	53	115	19	Magel2	23	68
10	Uniparental disomy	49	111	20	DNA methylation	22	63

## 4 Discussion

To our knowledge, this study is the first to use bibliometric analysis to examine the research knowledge mapping in the field of PWS from 2002 to 2022. An extremely high increasing trend of published articles was found in the last 5 years. The United States was the dominant country. University of Florida in USA published the largest proportion of papers on this topic. The majority of PWS articles were published in *American Journal of Medical Genetics Part A* and *Journal of Clinical Endocrinology & Metabolism*. Butler, Merlin G. and his colleagues have published 99 papers with 1,487 citations, highlighting their great influence in this field. The hotspots and frontiers will be genetic mechanism, diagnose, and emerging therapies in PWS research.

### 4.1 General information

The research on PWS can be broadly categorized into three time periods spanning from 2002 to 2022. In the initial phase (2002–2007), there was a steady increase in publication outputs, with twice as many papers published in 2007 compared to 2002. As illustrated in Figure 3d, the paper “Elevated plasma ghrelin levels in Prader Willi syndrome” by Cummings D.E. et al., published in Nature Medicine, experienced a surge in citations, starting in 2003 and ending in 2007. Concurrently, as shown in Figure 4d, the frequency of the keyword “circulating ghrelin” increased dramatically between 2003 and 2007. The ghrelin appears to have garnered significant interest from the research community, which has contributed to the upswing in publications on the topic. However, from 2008 to 2016, the publications have entered a plateau period. Over the past 5 years, there has been a rapid resurgence in papers, with one and a half times more papers published in 2022 than in 2017. In 2013, the article “Truncating mutations of MAGEL2 cause Prader-Willi phenotypes and autism” by Christian P. Schaaf et al. was published in Nature Genetics, and its citation experienced a burst from 2015 to 2018 (Figure 3d). Additionally, as depicted in Figure 4d, the frequency of the keyword “MAGEL2” bursted from 2017 to 2022. The significant interest in MAGEL2 among researchers has led to a surge in the number of publications. This shows that research on PWS continues to receive substantial attention from scholars. So, significant advancements have made in the understanding of PWS over the past 20 years.

In visualization analysis of countries/regions, nodes with centrality values exceeding 0.1 are considered relatively crucial. The USA exhibits the highest centrality value (0.37), closely followed by the UK with a centrality value of 0.33 (Table S1), indicating their critical role as key intermediaries within the international cooperation network for PWS research. China ranks ninth in publication output, but its centrality is zero, indicating that despite China’s increasing interest in PWS research, there is still room for improvement in research quality to achieve greater impact. Especially since 2019, China experienced significant surge in citations. This is mainly because the Chinese government attaches great importance to the management of rare diseases and has released a series of documents such as the “First Batch of Rare Disease List in China,” covering 121 rare diseases.

The results demonstrate close collaboration between countries (Figures 2a and S2a) and institutions (Figures 2b and S2b) where leading nations exhibit extensive breadth and intensity of academic cooperation network. For example, the United States has established strong academic collaborations with multiple countries. Similarly, China also engages in academic cooperation with countries such as the USA and UK, among others. As for institutions, cooperative institutions are more manifested in international cooperation and exchange of findings. For example, the University of Florida and University of Kansas in the United States, which has published a large number of articles, has collaborated with multiple institutions in other countries. Considering that this disease is very rare, this robust international collaboration greatly facilitates advancements in the field of PWS.

According to the authors’ results, Butler, Merlin G. (99) contributed significantly to PWS research, followed by Grugni, Graziano (86), Hokken-Koelega, Anita C.S. (64), Tauber, Maithe (60), and Crino Antonino (51). Notably, Butler, Merlin G., a professor at the University of Kansas Medical Center specializing in Psychiatry & Behavioral Sciences and Pediatrics played a pivotal role in advancing knowledge about PWS. He was instrumental in identifying PWS as his primary research focus during his career. In the early 1980s, he first reported that a chromosome 15 deletion inherited solely from the father is responsible for this syndrome’s genetic cause [15]. Later on in that decade, he collaborated with researchers from Harvard University using advanced DNA technology to determine that approximately one-third of cases result from maternal disomy 15 when a mother passes two copies of chromosome 15 instead of one to her child with PWS [16].

In Table 1, *American Journal of Medical Genetics Part A* and *Journal of Clinical Endocrinology & Metabolism* have published the highest number of articles related to PWS and received significant citations within this field. Most top co-cited journals belong to Q1 category indicating their high impact factor within academia. The current hot topics revolve around etiology investigation, pathogenesis understanding, current therapies, and emerging treatment options along with management strategies. These findings help identify core journals within this research domain. It is evident that highly influential articles are predominantly sourced from high IF journal, suggesting that the research on PWS is considered worthwhile by academic worldwide.

### 4.2 Hotspots and frontiers

Keyword co-occurrence analysis can exhibit the distribution and development of research themes. As illustrated in Table 2, high occurrence frequency keywords included PWS (1,040), obesity (188), GH (94), genomic imprinting (61), UPD (49), hyperphagia (57), and behavior (28). Three main clusters were generated from this analysis. The research hotspots and emerging frontiers within PWS research can be summarized as follows.

#### 4.2.1 Genetics and genetic subtypes of PWS

The three main genetic subtypes are represented by paternal 15q11-q13 deletion, maternal UPD 15, and imprinting defect. Two proximal chromosome 15q11-q13 breakpoints including BP1 and BP2 along with a distal breakpoint BP3 appear to predispose to the typical deletions observed in PWS cases (about 65–70% prevalence) [17,18]. These typical deletions fall into two classes: Type I deletion involving BP1 which is larger and Type II deletion involving BP2 which is smaller; both types involve BP3 as a common breakpoint [19].

Another significant genetic cause accounting for approximately 25–35% cases involves maternal disomy 15 where both copies of chromosome 15 are inherited from the mother. These genes on chromosome 15 are typically epigenetic silenced. The presence of disomy in chromosome 15 may have an impact on fetal development and clinical outcomes during pregnancy. Most PWS cases resulting from maternal disomy 15 exhibit the heterodisomic form [1,2,13,20]. While most cases of PWS are sporadic in nature, some families may carry a defective error caused by an epimutation or incomplete processing of the imprint during germ cell meiosis from the father or due to a microdeletion at the DNA imprinting center. Microdeletion defects have been reported in approximately 3–5% of cases as a result of imprinting defects [21,22]. This microdeletion can be passed down from the paternal grandmother to the father and increase the risk of another child being born with PWS.

#### 4.2.2 Clinical aspects of PWS

Diagnostic criteria for PWS have been established and periodically revised according to clinical findings and presentations [4,23]. Formal genetic testing is still necessary to confirm a clinical diagnosis of PWS through DNA methylation analysis and detection of chromosome 15 deletions or other anomalies related to this chromosome [22].

Severe obesity is a prominent symptom observed in individuals with PWS; prevalence rates for overweight and obesity among children and adolescents range around 40% [24], while this percentage increases significantly between 80 and 90% during adulthood [25,26]. Therefore, another approach for classifying clinical characteristics is according to nutritional phases. Classically, two primary stages of nutritional development have been described: the initial phase after birth, characterized by failure to thrive with inadequate feeding and severe hypotonia, followed by a childhood period (around 18–36 months of age), where the main characteristic is hyperphagia leading to obesity [27,28]. If left uncontrolled, significant hyperphagia in individuals with PWS can result in morbid obesity and contribute to diabetes, cardiovascular or orthopedic problems, and even death [29].

#### 4.2.3 Role of ghrelin

Ghrelin, a 28-amino acid peptide, is identified as an agonist of the GH secretagogue receptor 1a (GHSR1a), which is a G protein–coupled receptor. Upon binding to GHSR1a, ghrelin activates the release of GH from pituitary cells [30]. There are two types of ghrelin in the circulation, acylated ghrelin (AG) and unacylated ghrelin (UAG) [31,32]. While AG is known for stimulating appetite and its diabetogenic and obesogenic functions [33–35], UAG is recognized for its protective effects on beta cells and muscle cells while improving glycemic control [36–40].

Given these opposing effects between AG and UAG levels, the ratio of AG to UAG levels (AG/UAG ratio) may be significant. Previous studies consistently confirmed total hyperghrelinemia in infants, children, and adults with PWS. One study found that the AG/UAG ratio was increased in children and young adults with PWS while UAG levels were similar to those of controls [41], another study showed high levels of circulating UAG but stable AG levels in infants until 48 months [42]. These studies indicate varying AG/UAG ratios during different nutritional phases. More recently it was shown that the transition from failure to thrive to excessive weight gain and hyperphagia in infants and children with PWS coincides with an increase in the AG/UAG ratio occurring before the onset of hyperphagia [43].

### 4.3 Treatment options of PWS

#### 4.3.1 GH

GH deficiency has been documented in most children case of PWS [44]. Despite the mechanism underlying GH deficiency associated with PWS is completely unknown, GH has been recommended as the primary drug treatment for PWS due to its beneficial effects, including improved body composition, normalized height, enhanced mobility, and potentially improved cognitive function in both children and adults [45,46]. As part of the current standard of care for PWS, it is advised that GH therapy be initiated in children with confirmed genetic diagnosis of PWS at the earliest possible stage [47]. Following attainment of final height, long-term use of GH therapy may help maintain optimal body composition [48].

#### 4.3.2 Therapeutic perspectives targeting the ghrelin in PWS

Livoletide, an amino acid peptide and stable UAG analogue, was found to be well tolerated during a European double-blind placebo-controlled phase II trial conducted over a period of 14 days. It demonstrated significant improvement in hyperphagia among patients with PWS [49]. The study treatment also resulted in notable reductions in waist circumference, fat mass, and postprandial glucose levels. However, no changes were observed in ghrelin levels among patients receiving livoletide. Nevertheless, the second phase II study involving livoletide did not show sustained significant effects on hyperphagia after 3 months compared to placebo. Therefore, the development program was discontinued by the company.

Ghrelin *O*-acyltransferase (GOAT) is an enzyme responsible for converting UAG into AG. The inhibition of GOAT can suppress AG production while increasing UAG levels to counteract orexigenic and adipogenic effects. A study indicated that GLWL-01, a GOAT inhibitor, is safe and well tolerated [50]. It demonstrated that this GOAT inhibitor effectively lowers AG levels while elevating UAG levels, respectively, among patients with PWS. However, no modifications were observed regarding patients’ eating behaviors, BMI, blood glucose, and total cholesterol. Nevertheless, in light of the roles of AG and UAG and the potential for combination therapies that include a GOAT inhibitor, it may be necessary to refine the application of this drug for treating PWS.

#### 4.3.3 Oxytocin and carbetocin

Oxytocin is a neuropeptide hormone that plays crucial roles in social interactions, social skills, food intake, anxiety, energy expenditure, maternal behaviors, and body weight regulation [51,52]. Patients with PWS have reduced numbers of oxytocin-producing neurons [53], which could contribute to their poor social judgment and emotional control. Although several clinical trials on oxytocin treatment in PWS have been conducted [54], its beneficial effects were inconsistent. The absence of consistent positive results may reflect differences in dosage and administration rather than indicating that oxytocin has no role in treating hyperphagia and behavioral problems associated with PWS phenotype [54]. Further studies are warranted to elucidate the role of oxytocin in treatment of PWS.

A study showed that carbetocin, an oxytocin receptor agonist, had positive effects on hyperphagia and other behaviors among 17 children with PWS compared to those receiving placebo for 2 weeks [55], and tolerance was good. If these findings are confirmed by future research studies, then oxytocin might become another hormone used as supplementation therapy for individuals with PWS.

#### 4.3.4 Diazoxide

Diazoxide, an ATP-sensitive K^+^ channel (K-ATP) agonist, has been reported to have the therapeutic effect on children and adults with PWS through insulin secretion from pancreatic β-cells, modulation of hypothalamic NPY, the most potent endogenous neuropeptide, and activation of K-ATP channels in adipocytes [56]. In this study, a new once-a-day formulation of diazoxide significantly improved hyperphagia and its associated food-related behaviors among patients with PWS. Although the effects of diazoxide on hyperphagia associated with PWS are not yet fully understood, current evidence suggests that further research attention should be given to diazoxide.

#### 4.3.5 Glucagon-like peptide- receptor agonist

Liraglutide is a short-acting daily preparation approved by the FDA and EMA for treating obesity in patients aged 12 years and older [57,58]. In a case study by Senda et al., the benefits of liraglutide therapy in PWS were first reported [59]. The study showed that liraglutide monotherapy for 1 year reduced elevated plasma ghrelin levels in PWS, leading to improvements in overeating, BMI, visceral fat, and glycemic control. A longitudinal study evaluated the efficacy of exenatide therapy for 6 months in overweight or obese young adults with PWS. It reported reduced appetite and improved HbA1c levels but no change in weight or BMI [60].

In summary, hyperphagia progressive weight often results in severe obesity, metabolic dysfunction, cardiorespiratory difficulties, and premature death [61]. Given the challenges of achieving a healthy BMI through dietary restriction alone, there is an urgent need for pharmacotherapies targeting hyperphagia and inducing weight loss in PWS. However, little understanding of the pathogenesis of hyperphagia and weight gain hinders the development of therapeutic approaches for PWS. Nevertheless, significant progress has been made recently in pharmacotherapeutic research on hyperphagia and obesity in PWS with several promising new drugs on the horizon.

### 4.4 Limitations

This study has several limitations. First, it only utilized data from SCI-Expanded WoSCC, which restricts its analysis scope by excluding potentially relevant papers from other fields related to PWS. Second, the evaluation was confined to English articles or reviews thus overlooking valuable contributions written in other languages. Third, the exclusion of recent publications in 2023 hampers the study’s capacity to capture contemporary advancements and emerging trends effectively. Fourth, the attention given to recent papers may underestimate due to their relatively low citations. Finally, higher self-citation or regional focus might cause inaccurate conclusions.

## 5 Conclusion

The present study analyzed and visualized the research status of PWS over the past two decades. We found that the number of publications related to PWS shows an increasing trend over time. The study offered valuable assistance for scholars seeking core literature, potential research partners, journal publication decisions as well as research hotspots and frontiers in this field. Moreover, current therapeutic approaches for PWS are nonspecific and only partially satisfactory. Thereby, it is crucial to deepen and strengthen cooperation among countries and institutions in order to better understand this rare disease to address several fundamental questions such as the relationship between genetic defects and various phenotypes, and find effective therapeutic approaches.

## Supplementary Material

Supplementary material
