# Supplementary material for: A bibliometric analysis of Prader-Willi syndrome from 2002 to 2022
Source: Open Med (Wars). 2024 Nov 28;19(1):20241058. doi: 10.1515/med-2024-1058 (PMC11627057; doi:10.1515/med-2024-1058)
Supplement: Supplementary material [file med-2024-1058-sm.pdf]

Supplementary material

Table S1: The top 10 productive countries/regions and institutions

Rank	Country/region	Count	Centrality	Institution	Country/region	Count	Citations
1	USA	622	0.37	University of Florida	USA	101	4405
2	UK	204	0.33	University of Kansas	USA	79	2953
3	Italy	185	0.09	University of Alberta	Canada	69	1936
4	Netherlands	158	0.03	University of Cambridge	UK	59	3267
5	France	150	0.14	Dutch Growth Research Foundation	Netherlands	46	1150
6	Germany	124	0.11	Vanderbilt University	USA	45	1605
7	Canada	120	0.06	Bambino Gesu Children'S Hospital	Italy	42	919
8	Japan	120	0	Erasmus University Rotterdam	Netherlands	41	1782
9	Peoples r China	93	0	Erasmus Medical Center	Netherlands	39	650
10	Australia	92	0.02	Baylor College of Medicine	USA	35	1969

Table S2: Top 10 productive authors and co-cited authors

Rank	Author	Count	Co-cited Author	Citations
1	butler, merlin g.	99	butler, merlin g.	1487
2	grugni, graziano	86	cassidy, sb	1149
3	hokken-koelega, anita c. s.	64	dykens, em	730
4	tauber, maithe	60	goldstone, ap	715
5	crino, antonino	51	eiholzer, u	595
6	holland, anthony	48	miller, jennifer,l	533
7	miller, jennifer l.	48	holm, va	508
8	driscoll, daniel j.	46	carrel, al	377
9	wevrick, rachel	38	nicholls, rd	369
10	hoybye, charlotte	34	hoybye, charlotte	358

Table S3: The top 10 most cited references

Rank	Co-cited counts	Author	Year	Source	Title	Doi
1	159	Cassidy SB	2012	Genet Med	Prader-Willi syndrome	10.1038/gim.0b013e31822bead0
2	105	Angulo MA	2015	J Endocrinol Invest	Prader-Willi syndrome: a review of clinical, genetic, and endocrine findings	10.1007/s40618-015-0312-9
3	90	Cassidy SB	2009	Eur J Hum Genet	Prader-Willi syndrome	10.1038/ejhg.2008.165
4	80	Goldstone AP	2008	J Clin Endocr Metab	Recommendations for the Diagnosis and Management of Prader-Willi Syndrome	10.1210/jc.2008-0649
5	69	Goldstone AP	2004	Trends Endocrin Met	Prader-Willi syndrome: advances in genetics, pathophysiology and treatment	10.1016/j.tem.2003.11.003
6	67	Sahoo T	2008	Nat Genet	Prader-Willi phenotype caused by paternal deficiency for the HBII-85 C/D box small nucleolar RNA cluster	10.1038/ng.158
7	66	Butler MG	2019	Curr Pediatr Rev	Prader-Willi Syndrome - Clinical Genetics, Diagnosis and Treatment Approaches: An Update	10.2174/1573396315666190716120925
8	55	Butler MG	2019	J Med Genet	Molecular genetic classification in Prader-Willi syndrome: a multisite cohort study	10.1136/jmedgenet-2018-105301
9	52	Miller JL	2011	Am J Med Genet A	Nutritional Phases in Prader–Willi Syndrome	10.1002/ajmg.a.33951
10	49	Burnett LC	2017	J Clin Invest	Deficiency in prohormone convertase PC1 impairs prohormone processing in Prader-Willi syndrome	10.1172/JCI88648

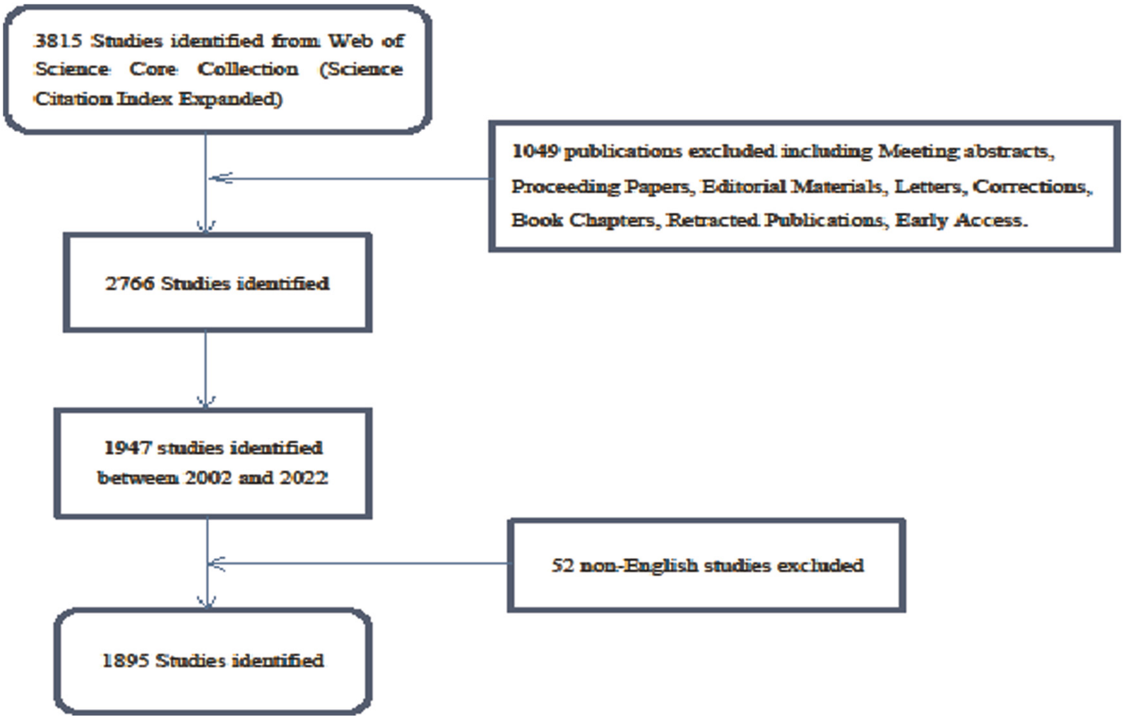

Figure S1: Flow chart of the search strategy.

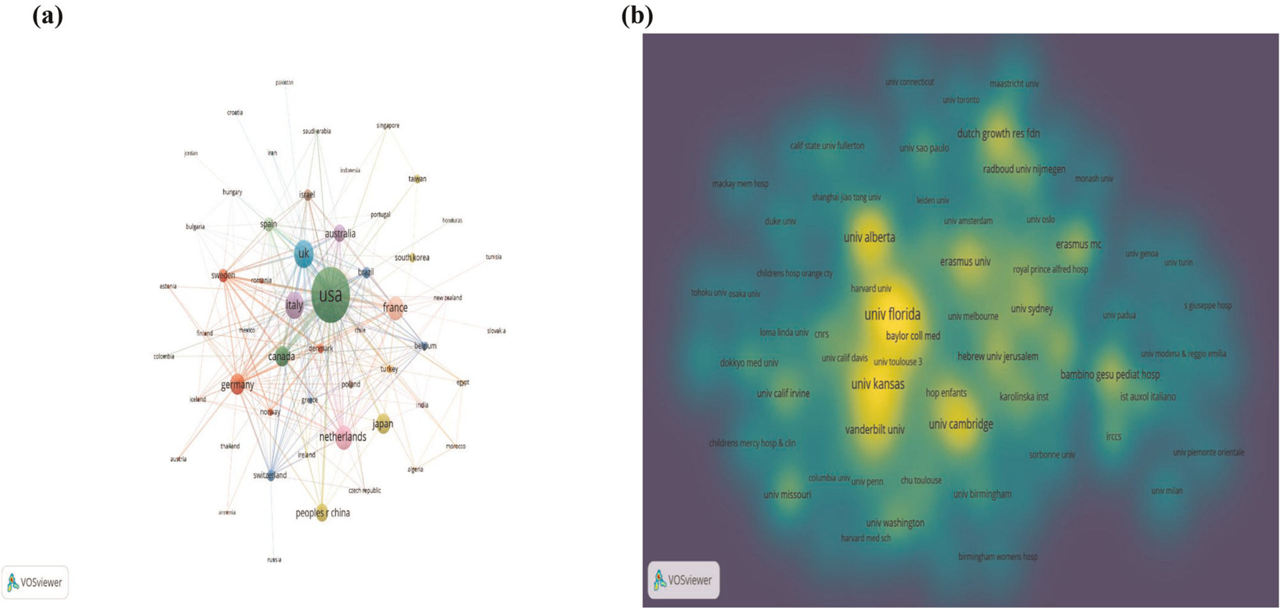

Figure S2: (a) Collaboration networks between countries/regions. (b) Institutions collaboration networks.

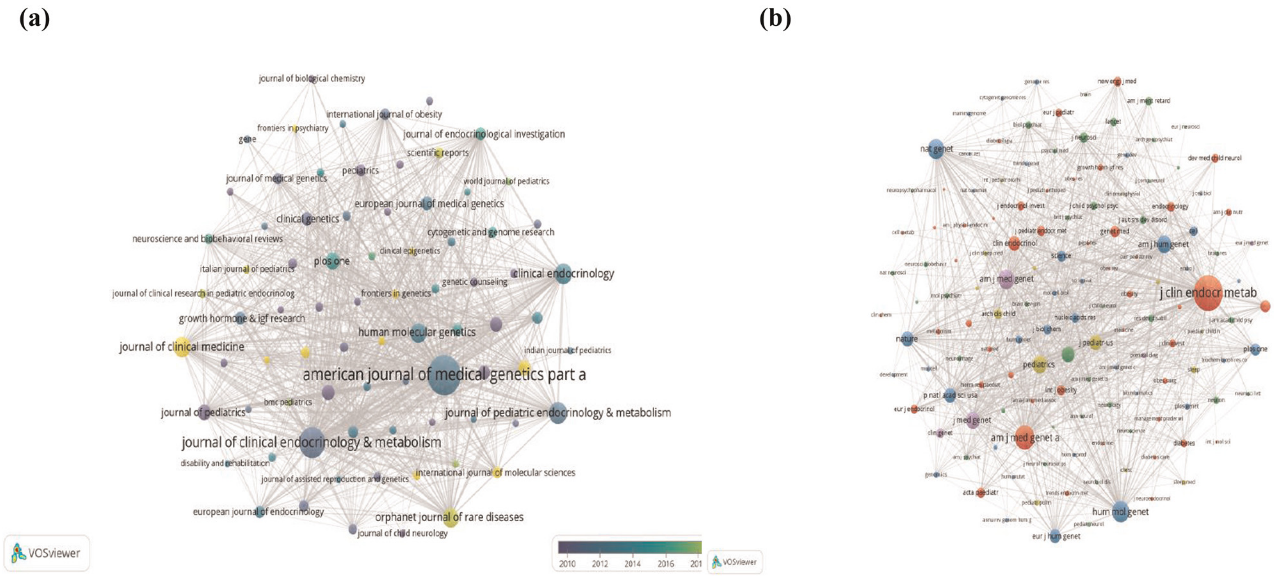

Figure S3: (a) Networks showing journals (b) co-cited journals.

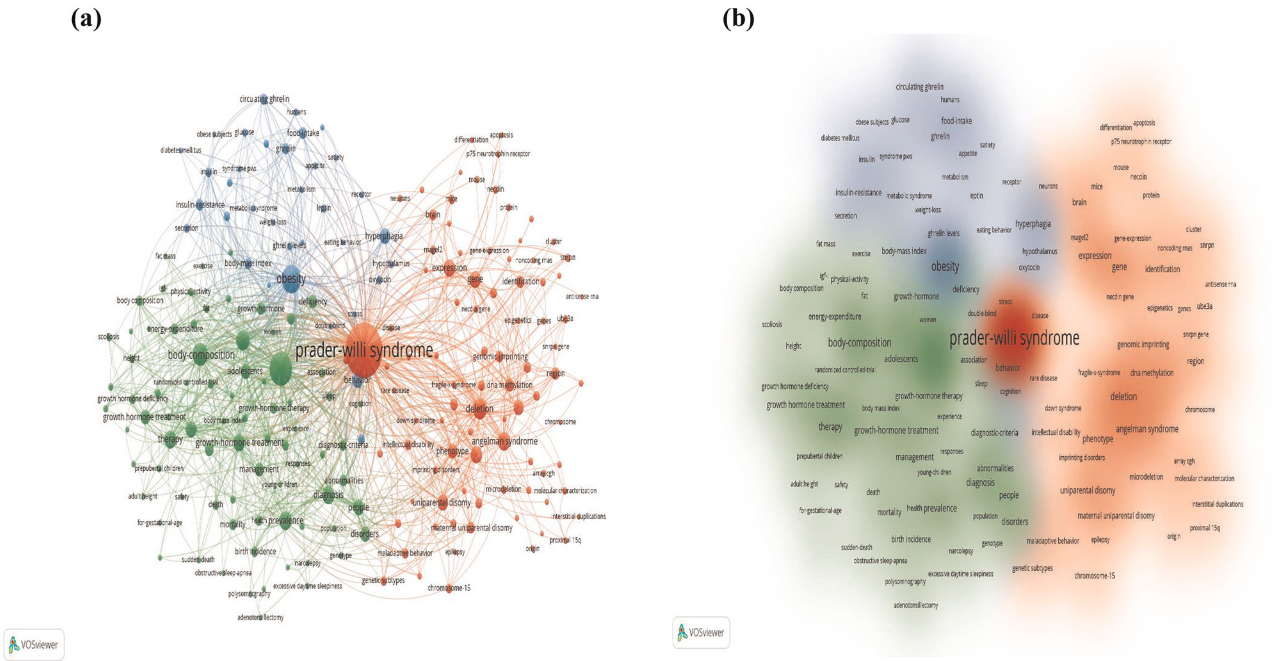

Figure S4: (a) All keywords co-occurrence networks. (b) All keywords cluster density visualization.

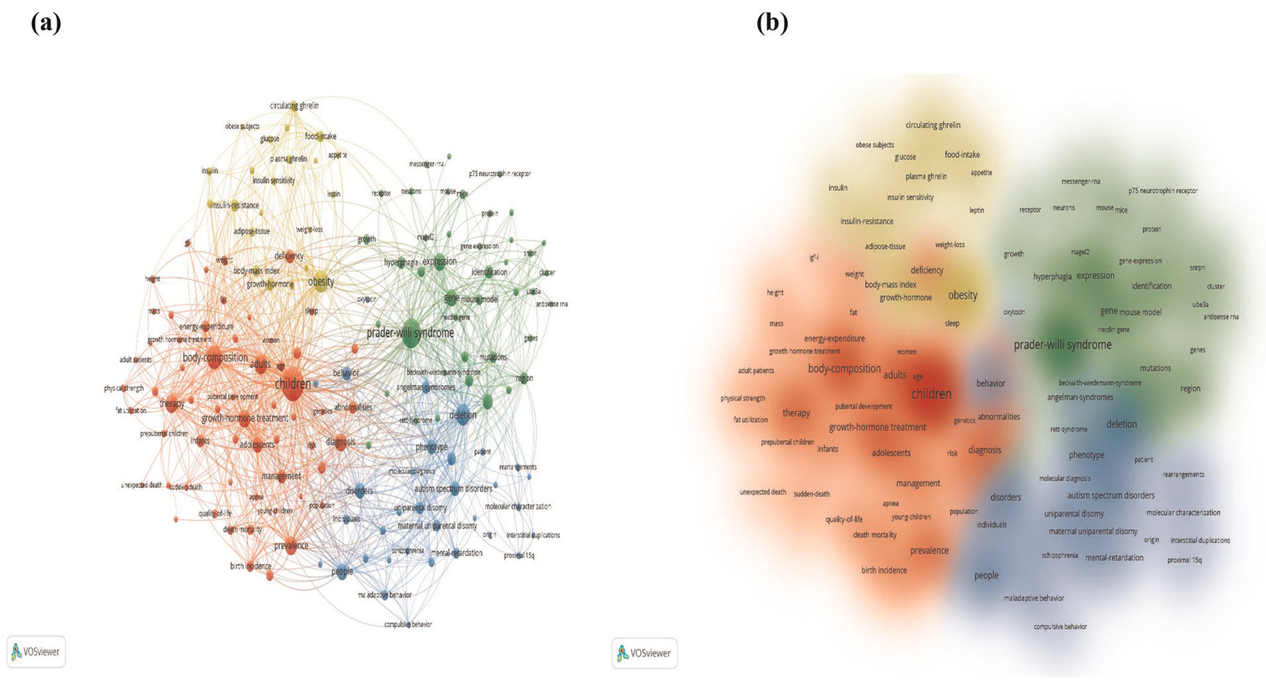

Figure S5: (a) Keywords plus co-occurrence networks. (b) Keywords plus cluster density visualization.
